# Targeting Follistatin like 1 ameliorates liver fibrosis induced by carbon tetrachloride through TGF-β1-miR29a in mice

**DOI:** 10.1186/s12964-020-00610-0

**Published:** 2020-09-15

**Authors:** Xin-Yi Xu, Yan Du, Xue Liu, Yilin Ren, Yingying Dong, Hong-Yu Xu, Jin-Song Shi, Dianhua Jiang, Xin Xu, Lian Li, Zheng-Hong Xu, Yan Geng

**Affiliations:** 1 School of Pharmaceutical Sciences, Jiangnan University, Wuxi, 214122 China; 2 National Engineering Laboratory for Cereal Fermentation Technology, Jiangnan University, Wuxi, Jiangsu China; 3 Jiangsu Engineering Research Center for Bioactive Products Processing Technology, Jiangnan University, Wuxi, 214122 P.R. China; 4 State Key Laboratory of Medicinal Chemical Biology, College of Life Sciences, Nankai University, Tianjin, 300071 China; 5 Department of Medicine, Cedars-Sinai Medical Center, Los Angeles, CA 90048 USA; 6 Cambridge-Suda Genomic Resource Center, Soochow University, Suzhou, 215123 China; 7 Wuxi No. 2 People’s Hospital, Wuxi, 214002 China

**Keywords:** Hepatic fibrosis, Cell differentiation, Transforming growth factor-β (TGF-β) signaling, Follistatin like 1 (Fstl1), microRNA

## Abstract

**Background:**

Hepatic fibrosis is a pathological response of the liver to a variety of chronic stimuli. Hepatic stellate cells (HSCs) are the major source of myofibroblasts in the liver. Follistatin like 1 (Fstl1) is a secreted glycoprotein induced by transforming growth factor-β1 (TGF-β1). However, the precise functions and regulation mechanisms of Fstl1 in liver fibrogenesis remains unclear.

**Methods:**

Hepatic stellate cell (HSC) line LX-2 stimulated by TGF-β1, primary culture of mouse HSCs and a model of liver fibrosis induced by CCl4 in mice was used to assess the effect of Fstl1 in vitro and in vivo.

**Results:**

Here, we found that Fstl1 was significantly up regulated in human and mouse fibrotic livers, as well as activated HSCs. Haplodeficiency of *Fstl1* or blockage of Fstl1 with a neutralizing antibody 22B6 attenuated CCl_4_-induced liver fibrosis in vivo. Fstl1 modulates TGF-β1 classic Samd2 and non-classic JNK signaling pathways. Knockdown of Fstl1 in HSCs significantly ameliorated cell activation, cell migration, chemokines C-C Motif Chemokine Ligand 2 (CCL2) and C-X-C Motif Chemokine Ligand 8 (CXCL8) secretion and extracellular matrix (ECM) production, and also modulated microRNA-29a (miR29a) expression. Furthermore, we identified that Fstl1 was a target gene of miR29a. And TGF-β1 induction of Fstl1 expression was partially through down regulation of *miR29a* in HSCs.

**Conclusions:**

Our data suggests TGF-β1-miR29a-Fstl1 regulatory circuit plays a key role in regulation the HSC activation and ECM production, and targeting Fstl1 may be a strategy for the treatment of liver fibrosis.

Video Abstract

**Graphical abstract:**

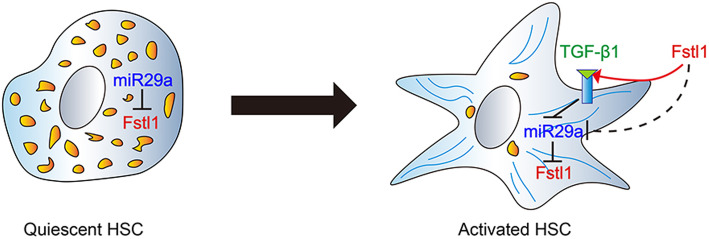

## Background

Liver fibrosis is a scarring process that occurs in most chronic liver diseases, including nonalcoholic fatty liver disease (NAFLD), alcoholic liver disease, and hepatitis B/C virus infection. Hepatic stellate cells (HSCs), liver specific mesenchymal cells, are the primary cell type responsible for the development of liver fibrosis. HSCs contain numerous lipid droplets in normal liver. During chronic liver injuries, HSCs are activated by cytokines and chemokines from damaged hepatocytes and immune cells, then transdifferentiate to myofibroblasts that produce massive ECM and fibrogenic cytokines [1, 2]. The activated HSCs, in turn, release more cytokines and chemokines, leading to enhanced inflammatory responses in injury area [3]. Among many cytokines mediating the fibrotic cascades, transforming growth factor-β (TGF-β) is a central profibrotic growth factor [4]. TGF-β plays a key role in HSCs activation, migration and transdifferentiation into myofibroblasts, as well as simulating the synthesis of ECM [5, 6]. Hepatic specific overexpression of mature TGF-β1 leads to liver fibrosis in mice [7]. Blocking TGF-β1 signaling pathway by TGF-β1 antibodies or antisense oligonucleotides, and soluble TβRII attenuated liver fibrosis in experimental models [8].

MicroRNAs (miRNAs) are endogenous 20–22 nucleotides RNAs that control translation and transcription of many genes. MicroRNA-29 families (*miR-29a/b/c*) [9] are known to be the downstream target of TGF-β and play fundamental roles in liver [10, 11], lung [12, 13], and cardiac fibrosis [14]. Members of *miR-29* family were down regulated in HSCs activation in vitro and in fibrotic livers in human and mice [10, 11, 15]. Moreover, patients with liver fibrosis showed significantly lower levels of circulating *miR-29a*, when compared with healthy controls [10]. Ectopic expression of *miR-29b* in the liver of mice attenuated CCl_4_ induced liver fibrosis [11]. However, mechanism of action of *miR-29a* in liver fibrosis remains largely unclear.

Follistatin-like 1 (Fstl1) is a secreted glycoprotein belonging to the Follistatin (Fst) family and secreted protein acidic rich in cysteines (SPARC) family [16], which can be induced by TGF-β [17]. Although Fst expression was unchanged in activated HSCs, Fst treatment ameliorated early liver fibrosis in experimentally induced liver fibrosis in rats by blocking Activin bioactivity [18]. SPARC expression in hepatic tissue was significantly increased during the development of liver fibrosis, and targeting SPARC through an adenovirus carrying antisense SPARC suppressed HSCs activation in thioacetamide induced liver fibrosis in rats [19]. As the smallest member in the Fst-SPARC family, the role of Fstl1 in liver fibrosis and its therapeutic potential has not been fully investigated.

Homozygous *Fstl1*^*−/−*^ mice die of respiratory failure shortly after birth [20], so *Fstl1*^*+/−*^ or conditional knockout mice have been used to study the lung and kidney fibrosis [21, 22]. The results showed that haplodeficiency of *Fstl1* or blockage of Fstl1 with a neutralizing antibody attenuated bleomycin induced lung fibrosis in mice [21]. Cardiac-specific Fstl1-deficient mice promoted tubulointerstitial fibrosis after subtotal renal ablation compared with wild-type mice [22]. In addition, application of the human FSTL1 protein via an epicardial patch stimulates pre-existing cardiomyocytes proliferation, improves cardiac function and attenuated fibrosis in animal models of myocardial infarction [23]. Northern blot analysis of murine tissues showed there was barely any Fstl1 transcript in the liver [24]. Recently, Fstl1 was identified as a fibrosis modifier by in vivo siRNA silencing screen [25]. Knockdown Fstl1 suppressed HSCs activation [26]. These data indicate that the role of Fstl1 in tissue fibrosis is controversial.

RNA deep sequencing and function assays revealed that *FSTL1* may be an endogenous target of *miR-29a* in human myotubes [27]. MiR-29a can promote the neurite outgrowth by targeting extracellular matrix related genes including Fstl1 [28]. In this study, we aim to analyze the role of Fstl1 in liver fibrosis by using TGF-β1 activated HSCs in vitro and a mouse model of CCl_4_-induced liver fibrosis. We found that Fstl1 is evolved in the pathogenesis of liver fibrosis through a TGF-β1-*miR29a*-Fstl1 regulatory circuit and can serve as a therapeutic target for the treatment of liver fibrosis.

## Methods

### Chemicals and reagents

CCl_4_ and Olive Oil were from Sigma-Aldrich (St Louis, MO, USA). Fstl1 neutralizing antibody was generated as described previously [21]. Fstl1 siRNA and scramble RNA were purchased from Genechem Company (Shanghai, China). The mimics and inhibitor of miR29a were purchased from Ruibo Company (Guangdong, China). The α-SMA, GAPDH antibodies were purchased from Santa Cruz Biotechnology (USA). The Fstl1 antibodies were purchased from Santa Cruz Biotechnology (USA) or R&D systems (USA). Smad2, p-Smad2, p-JNK and JNK were purchased from Cell Signaling Technology (USA).

### Subjects

The study was approved by the Institutional Review Board of Wuxi No.2 People’s Hospital (No. 20170608) and were in accordance with the principles of the Declaration of Helsinki as revised in 2000. The study includes 27 healthy controls and 19 patients (Table S1). All participants signed a written consent form before entering the study. All patients included in this study were diagnosed according to the respective diagnostic criteria. The healthy volunteers were recruited from the medical examination center of Jiangnan University that had normal aminotransferase activities, no history of liver disease or alcohol abuse and tested negative for HBV, HCV and HIV infections. Paraffin liver sections (LV1201) were from Alenabio.com (Xi’an, China). All human tissues are collected under IRB and HIPPA approved protocols, and approved for commercial product development.

### Enzyme-linked immunosorbent assay (ELISA)

The amount of FSTL1 (Cloud-Clone, USA) in serum and C-C Motif Chemokine Ligand 2 (CCL2) (Sino Biological, Beijing) or C-X-C Motif Chemokine Ligand 8 (CXCL8) (Invitrogen, USA) released from LX-2 cells into the culture medium was determined using commercially available ELISA kits according to the manufacturer’s instructions. Recombinant standards of FSTL1, CCL2 or CXCL8 provided in the kit and the serum or isolated culture medium were added to a plate pre-coated with a monoclonal antibody against the chemokine. After incubation for 1 h, the plate was washed and incubated with an enzyme-linked polyclonal antibody specific for FSTL1, CCL2 or CXCL8. After several washes, the substrate solution was added, and the color intensity was measured. A standard curve was used for determination of the amount of FSTL1, CCL2 or CXCL8 present in the samples.

### Animal model of liver fibrosis and treatment

The Animal Research Committee of Jiangnan University and Nankai University approved all animal experiments. Male C57BL/6 or BABL/c mice at 8 weeks were purchased from Shanghai Slac Laboratory Animal CO.LTD (China). *Fstl1*^*+/−*^ mice were described previously [20] and backcrossed to C57BL/6 background for more than ten generations. The mice were allowed free access to tap water and a chow diet (M01-F25–20150922034, Shanghai SLAC Laboratory Animal Co., Shanghai, China). Liver fibrosis was induced by intraperitoneal (i.p.) injection of 0.5 ml/kg CCl_4_ (25% solution in olive oil) twice per week [29]. At designated time points after CCl_4_ or olive oil injection, mice were euthanized with phenobarbital sodium by i.p. injection, and livers were harvested for further analyses. Fstl1-neutralizing antibody (clone 22B6) or its control isotype antibody (IgG1) was intravenously injected (25 μg/mouse/each time) along with CCl_4_ treatment. The mouse livers were harvested 28 d after CCl_4_ injury. Tissues were sectioned for Picro-sirius red (PSR) staining to assess the degree of fibrosis. Collagen contents in the liver were measured with a conventional hydroxyproline method [30].

### Primary hepatic stellate cells isolation, cell culture and drug treatment

The isolation of HSCs from murine livers can be divided into three main sequential stages [31]. Briefly, the mouse livers were in situ perfused with pronase and collagenase. And then, the liver tissues were carefully removed, minced under sterile conditions and further digested with pronase/collagenase. At last, the HSCs were isolated with density gradient–based separation from other hepatic cell populations.

Human HSCs cell line LX-2 cells, rat HSCs cell line CFSC-8B and HSC-T6 were obtained from the cell bank of Xiangya Central Experiment Laboratory of Central South University (Changsha, China). Cells were cultured in DMEM or RPMI 1640 supplemented with 10% heat-inactivated FBS (Gibco, USA) and antibiotics at 37 °C in a humidified atmosphere of 5% CO_2_. Cells were grown to 100% confluence and serum starved for 24 h before treatment. Cells were pretreated with 2 μg/ml antibody (22B6) or control IgG1 for 24 h and then treated with 5 ng/ml TGF-β1. Fstl1 siRNA (40 nM), scramble control RNA (40 nM), *miR29*a mimics (100 nM) or inhibitors (200 nM) were transiently transfected into LX-2 cells using Lipofectamine RNAi max (Invitrogen, CA, USA) for 48 h.

### RNA isolation and qRT-PCR analysis

Total RNA was extracted from mouse liver tissue or cells with Trizol reagent (Invitrogen, CA, USA). We performed RNA isolation and qRT-PCR analysis as previously described [23]. Gene expressions were measured relative to the endogenous reference gene *Gapdh* using the comparative CT method and the sequences of specific primer pairs for *Fstl1*, *α-SMA*, and *Col1a1* were described previously [21]. The expression level of mature *miR-29a* was quantified by TaqMan microRNA assays (Mm04238191_s1, Applied Biosystems, CA, USA).

### Western blot analysis

Cells or liver tissues were washed with ice cold DPBS and re-suspended in RIPA buffer with protease inhibitor (Sigma-Aldrich, MO, USA). Protein was resolved by SDS-polyacrylamide gel electrophoresis and transferred to PVDF membranes. After blocking, they were probed with primary antibodies overnight at 4 °C, then incubated with horseradish peroxidase-conjugated secondary antibody for 1 h at room temperature. The bands were visualized using ECL reagents (Thermofisher Scientific, USA). Band intensity on scanned films was quantified using Image lab software (Bio-Rad Laboratories, Inc. USA). The ratio of the relevant protein was subjected to internal control (GAPDH).

### Cell migration assays

The cell migration assay was performed with Transwell chambers with 8-μm pores (Corning, USA). LX-2 or primary mouse HSCs (2.5 × 10^4^ cells per chamber) in serum free medium were plated in the upper chambers in duplicate filters. DMEM containing 10% heat-inactivated FBS was added to the lower chamber as a chemoattractant. After 24 h, non-migrating cells were removed from the upper surface, and filters were stained with crystal violet. Migrated cells were counted in five representative microscopic fields (100× magnification).

### Measurement of serum aminotransferase activities

The activities of alanine aminotransferase (ALT) and aspartate aminotransferase (AST) in serum were estimated spectrophotometrically using commercial diagnostic kits (Jiancheng Institute of Biotechnology, Nanjing, China).

### Luciferase reporter assay

The luciferase reporter assay was conducted using a Dual-Luciferase Reporter Assay System (Beyotime Biotechnology, China). PmiR-RB-Report™ vector is specially used to identify direct targets of microRNA. The Wildtype (WT) or mutant (Mut) 3 ‘UTR region of Fstl1 was cloned to the downstream of reporter Renilla luciferase gene (hRluc) by XhoI and NotI digestion. The primers for clone Fstl1-WT were 5’-GCG GCT CGA GGC AAT AAA GGA TAT GAA GGT GGC T-3′ and 5′- AAT GCG GCC GCA TGA AGT GGT GGG ACT ACT GAA AA-3′. The primers for clone Fstl1-Mut were 5′- TTA CCA AAC CAC GAT TTT CTC TGT AAA ACA CTT-3′ and 5′- CAG AGA AAA TCG TGG TTT GGT AAA AAG TAT TTT-3′. LX-2 cells (8.0 × 10^3^/well) were seeded into 96-well plates for 24 h and then the cells were transiently co-transfected with pmiR-RB-Report™-Fstl1-WT/−Mut plasmids and miR29a mimics using Lipofectamine 3000 (Thermofisher, USA). Cells were lysed and assayed for Renilla luciferase activity 48 h after transfection. 100 μl cell extracts were subjected to the Dual Luciferase Reporter Gene Assay Kit in Multiscan Spectrum. The firefly luciferase (hLuc) was used as internal control.

### Statistical analysis

Data are expressed as means ± SEM. Differences in measured variables between experimental and control groups were assessed by using Student’s test. Differences in multiple groups were assessed by using one-way analysis of variance (ANOVA), and the Tukey test was used for determining the significance. Results were considered statistically significant at *P* < 0.05. All analyses were conducted in Graphpad Prism software version 7.0.

## Results

Over-expression of FSTL1 in Serum and Livers of Human Patients with Chronic liver diseases.

**Fig. 1:**
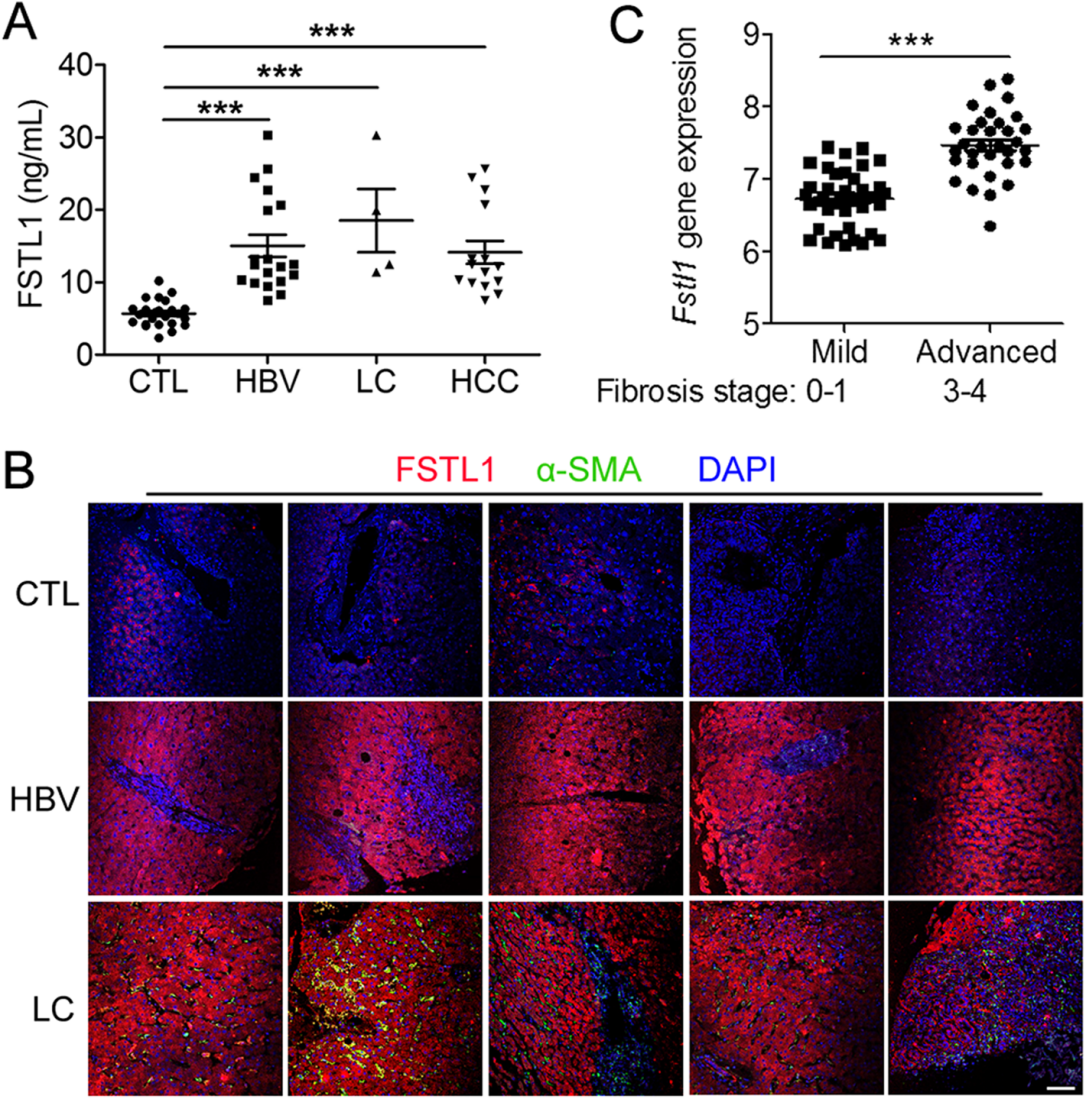
FSTL1 expression in chronic liver diseases. **a** Serum concentrations of FSTL1 in healthy CTL (*n* = 27) and patients with HBV (*n* = 19), LC (*n* = 4) and HCC (*n* = 15). **b** Paraffin liver sections from healthy CTL, and patients with HBV and LC were stained with FSTL1 (red), α-SMA (green) and DAPI (*n* = 5 per group). Scale bar: 100 μm. HBV, Viral Hepatitis B; LC, Cirrhosis; HCC, hepatocellular carcinoma. **c** *FSTL1* expression in human liver tissues was examined in a published gene-profiling dataset (GEO: GSE31803). The degree of liver fibrosis is divided into mild (*n* = 40) and severe forms (*n* = 32). Throughout, data represent mean ± SEM. ***P < 0.001

Serum concentrations of FSTL1 levels were determined for healthy controls (CTL) and patients with viral hepatitis B (HBV), cirrhosis (LC) and hepatocellular carcinoma (HCC). The result showed that FSTL1 levels were higher in patient groups than those in CTL (Fig. 1a, P < 0.05 and Table S1). FSTL1 immunostaining was weak in liver sections from CTL and was increased significantly (more than two-fold, *p* < 0.05) in the cytoplasm of hepatocyte in liver sections from patients with HBV and LC which co-stained with α-SMA (Fig. 1b). FSTL1 protein was also found aberrantly increased in HCC tissues compared to adjacent liver tissues [32]. Then we analyzed *FSTL1* expression in a gene-profiling dataset of percutaneous liver biopsies from NAFLD patients [33] through The NCBI Gene Expression Omnibus (GEO accession:GSE31803). Clinicians rely upon the severity of liver fibrosis to segregate patients with NAFLD into sub-population at low versus high-risk for eventual liver-related morbidity and mortality. There was a significance increase in *Fstl1* mRNA expression in liver tissues of high-risk (fibrosis stage 3–4) compared with low-risk (fibrosis stage 0–1) (Fig. 1c). These data indicate that FSTL1 may contribute to the progression of chronic liver diseases.

### Pathological expression of FSTL1 in fibrotic livers in CCl_4_-injured mice

**Fig. 2:**
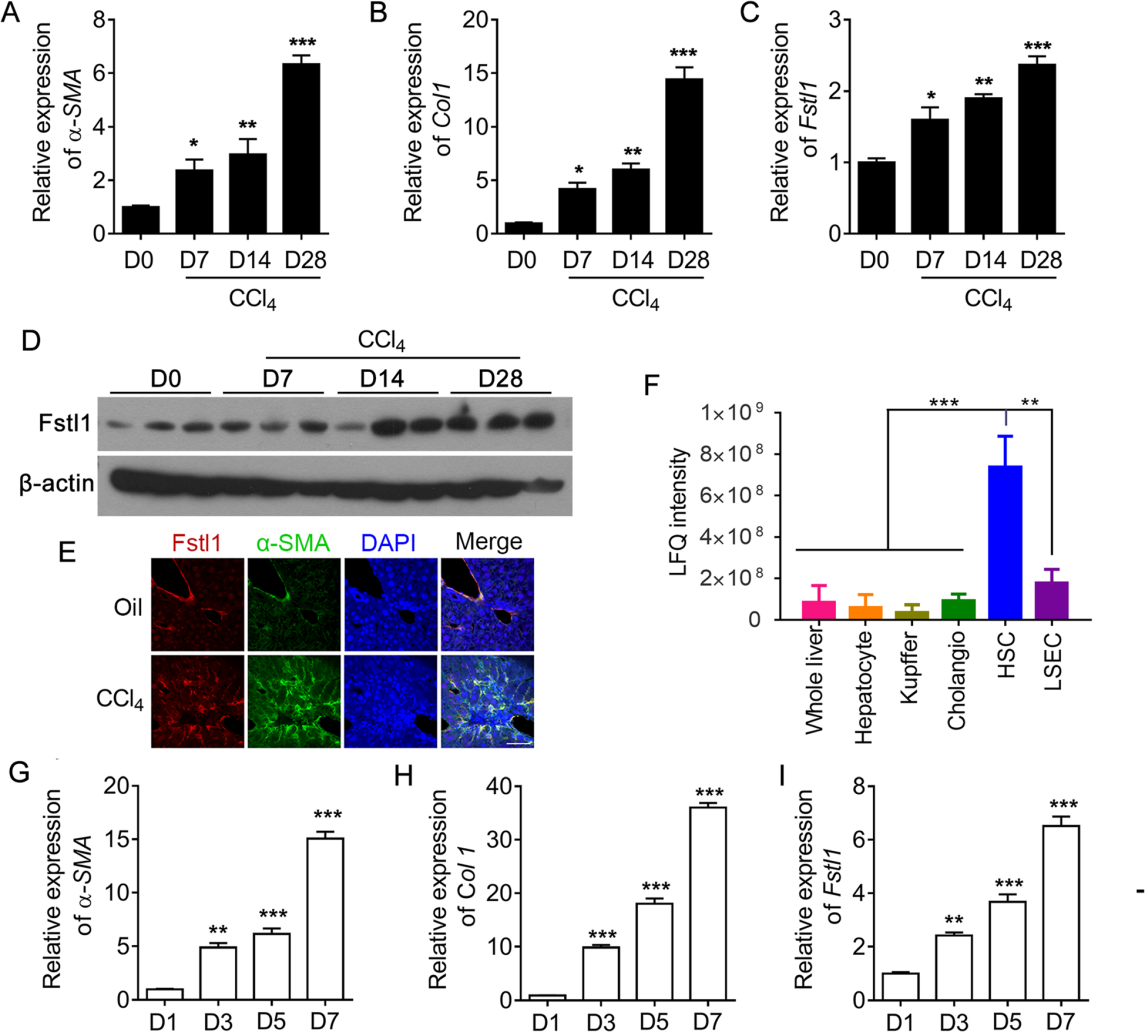
Fstl1 was associated with liver fibrosis and the activation of hepatic stellate cells. **a-c** qRT-PCR analysis of *α-SMA*, *Col1*, *Fstl1* mRNA expression in liver tissues of C57BL/6 mice at the indicated time points after CCl_4_ treatment (*n* = 6 per group). **d** Western blot analysis of Fstl1 protein in liver tissues of C57BL/6 J mice at the indicated time points after CCl_4_ treatment (*n* = 3 per group). β-Actin was used as a loading control. **e** Frozen liver sections from oil or CCl_4_ treated mice were stained with Fstl1 (red), α-SMA (green) and DAPI (blue). Scale bar: 25 μm. **f** LFQ (label-free protein quantification) intensity of Fstl1 in murine liver, hepatocyte, kupffer, cholangio, HSC and LSEC (liver sinusoidal endothelial cells) (n = 4 per group). **g-i** qRT-PCR analysis of *α-SMA*, *Col1*, *Fstl1* mRNA expression in primary isolated mouse HSCs in in vitro culture at the indicated time points (n = 5 per group). Throughout, data represent mean ± SEM.* *P* < 0.05, **P < 0.01, ***P < 0.001

Then we examined the expression of Fstl1 in a well-characterized murine model of CCl_4_ induced liver fibrosis. After 7, 14 and 28 days of repetitive CCl_4_ treatment, the gradually elevated expression of *α-SMA* and *Col1* suggested the persistent existence of liver damage and scar formation. CCl_4_-induced injury stimulated Fstl1 expressions at mRNA and protein levels significantly after 28 days (Fig. 2a-d). We detected Fstl1^+^ cells co-stained with HSCs activation marker α-SMA in mouse livers at 28 days after prolonged administration of CCl_4_ (Fig. 2e). Fstl1 was expressed higher levels in the HSCs than the other cell types in the liver, including hepatocyte, Kupffer cells, intrahepatic cholangiocytes and liver sinusoidal endothelial cells (LSECs) by Mass spectrometry-based proteomics (Fig. 2f) [34]. FSTL1 gene expression was significantly up-regulated (more than 5 fold) in human active cell line LX-2 compared with primary HSCs through DNA microarray analyses [35]. Furthermore, senescence of activated HSCs limits liver fibrosis [36]. Based on the GEO database (GEO accession: GDS3492), the gene expression of Fstl1 was significantly higher in growing activated HSCs than senescent HSCs stimulated by DNA damage drug etoposide (Figure S1). We also isolated the primary mouse HSCs (mHSCs) and confirmed the expression of *α-SMA* and *Col1* were up-regulated in in vitro culture. The expression level of *Fstl1* was gradually increased during this activation progress (Fig. 2g-i). These data suggest that Fstl1 is a fibrosis related gene and may be critical for the activation of HSCs.

### *Fstl1*^*+/−*^ mice have an attenuated fibrotic phenotype after liver injury

**Fig. 3:**
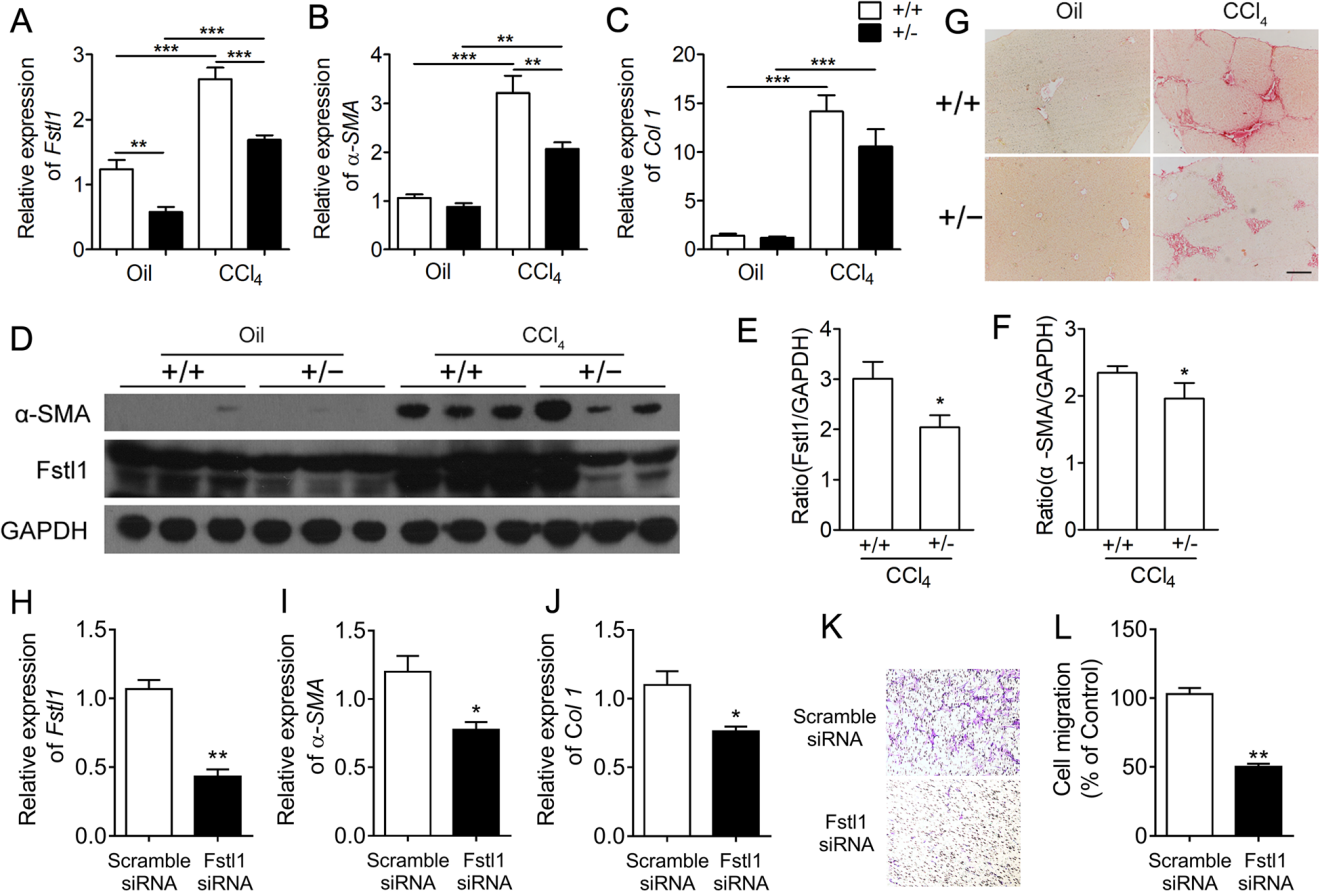
Targeting Fstl1 attenuated liver fibrosis in vivo and HSC activation in vitro. **a-c** *Fstl1*^*+/−*^ mice and littermate control mice were treated with oil or CCl_4_ for 28 days, and liver tissues were harvested for the following analyses. qRT-PCR analysis of *Fstl1*, *α-SMA* and *Col1*mRNA expressions (n = 5 per group). **d** Western blot analysis α-SMA and Fstl1 protein in liver tissues (*n* = 3 per group). **e-f** Band intensity was quantified using Image J software and expressed as relative intensity compared with control (*n* = 7 per group). The ratio of Fstl1, α-SMA were subjected to GAPDH. **g** Sirius red staining of liver sections. Scale bars: 100 μm. **h-j** Fstl1 siRNA (40 nM) transfection was performed on primary isolated mHSCs using Lipofectamine RNAimax. The gene expressions of *Fstl1*, *α-SMA*, *Col1* mRNA were assessed by qRT-PCR (n = 5 per group). **k-l** The migration of mHSCs was measured using the transwell system. Transmigration was evaluated 24 h after seeding the cells, by counting crystal violet-staining cells on the underside membrane by light microscopy (n = 5 per group). Images were photographed at 100 amplifications. Throughout, data represent mean ± SEM.* *P* < 0.05, **P < 0.01, ***P < 0.001

To determine the role of Fstl1 in vivo, we subjected *Fstl1*^*+/−*^ and littermate wild type (WT) mice to the CCl_4_ induced liver fibrosis. *Fstl1*^*+/−*^ mice had significant less *Fstl1* and *α-SMA* gene expression in fibrotic livers than that in the WT mice (Fig. 3a,b). The expression of *Col1* was also reduced in *Fstl1*^*+/−*^ mice, whereas did not reach statistical significance (Fig. 3c). The protein expression level of Fstl1 and α-SMA were downregulated in livers of *Fstl1*^*+/−*^ mice compared with WT mice (Fig. 3d-f). *Fstl1*^*+/−*^ mice also showed reduced degree of liver fibrosis, as determined by Sirius-red staining (Fig. 3g). These data indicate that Fstl1 is induced in response to liver injury and may promote live fibrosis in vivo.

### Silencing Fstl1 inhibits HSCs activation in vitro

To test whether TGF-β1 might regulate the expression of Fstl1 in HSCs, human HSC cell line LX-2, rat HSC cell lines CFSC-8B and HSC-T6 were used. Gene and protein expression of Fstl1 were increased by TGF-β1 in a time and dose dependent manner, correlating with increases in α-SMA and Col1 expression in these cells (Figure S2-S3). FSTL1 siRNA was highly effective in decreasing Fstl1 gene and protein expression relative to a scramble siRNA control (Figure S4a-b). Decreased FSTL1 expression led to the inhibition of expression of α-SMA and COL1 (Figure S4a-c). In addition, knockdown FSTL1 inhibited phosphorylation of JNK and TGF-β1 induced phosphorylation of Smad2 (Figure S4b-c). Chemokines CCL2 and CXCL8 have been shown to play critical roles for recruitment of inflammatory cells and their expression has been linked to liver fibrosis [37–39]. TGF-β1 significantly up regulated CCL2 and CXCL8 concentrations in cell culture medium, whereas si-FSTL1 inhibited their expression (Figure S4d-e). Importantly, we confirmed that knockdown Fstl1 significantly depressed the expression of *α-SMA* and *Col1* and decreased the cell migration in primary culture of mouse HSCs (Fig. 3h-l). Therefore, these data demonstrated that Fstl1 promotes the activation and transdifferentiation of HSCs in vitro.

### Blocking Fstl1 signaling attenuates CCl_4_ induced liver fibrosis in mice and inhibited TGF-β1 activated HSCs in vitro

**Fig. 4:**
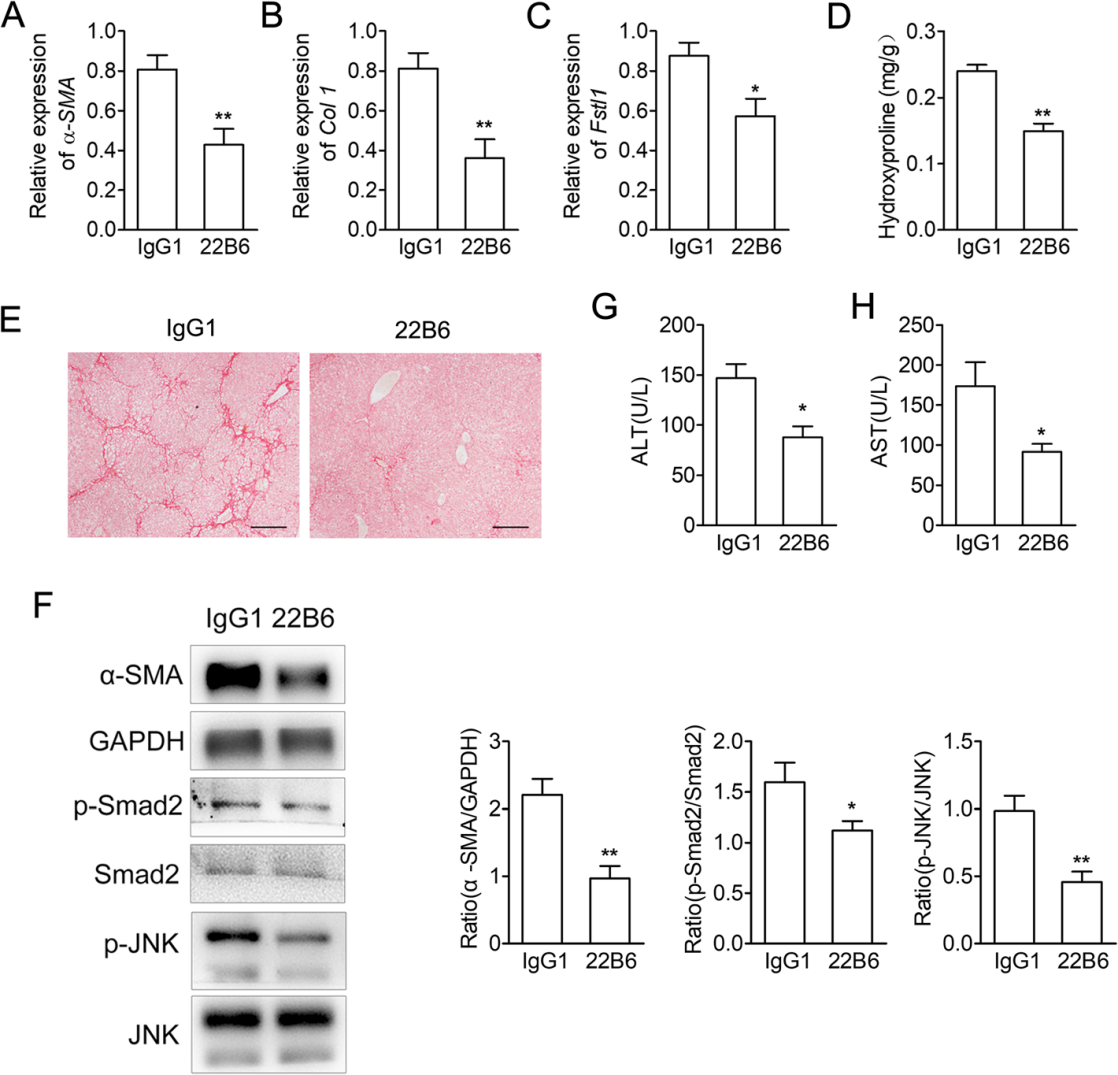
Fstl1-neutralizing antibody attenuates CCl_4_-induced liver fibrosis. **a-c** Babl/c mice were subjected to CCl_4_ along with Fstl1-neutralizing antibody 22B6 or isotype control IgG1 (25 μg/mouse) twice a week, and livers were harvested 4 weeks later for the following analyses (n = 6 mice per group). qRT-PCR analysis of *α-SMA*, *Col1* and *Fstl1* mRNA expressions. **d** Hydroxyproline contents in liver tissues were measured. **e** Sirius red staining of liver sections. Scale bars, 100 μm. **f** Protein expression levels of α-SMA, p-Smad2, Smad2, p-JNK, JNK in liver tissues were assessed by Western blot. Band intensity was quantified using Image J software and expressed as relative intensity compared with control. The ratio of α-SMA were subjected to GAPDH. The ratio of p-Smad2 was subjected to Smad2. The ratio of p-JNK was subjected to JNK. (G,H) Serum levels of ALT and AST were measured. Throughout, data represent means ± SEM. *P < 0.05, **P < 0.01

Then we examined whether Fstl1 neutralizing antibody (22B6 mAb) would ameliorate CCl_4_-induced liver fibrosis in vivo, we treated Babl/c mice with 22B6 mAb or IgG1 along with CCl_4_ treatment. After 28 days, 22B6 mAb treatment significantly down regulated gene expressions of *α-SMA*, *Col1* and *Fstl1* (Fig. 4a-c). 22B6 mAb treatment also prevented the development of fibrosis, compared with IgG1 treated mice, as determined by hydroxyproline content, α-SMA protein level and collagen staining (Fig. 4d-f). Serum ALT (alanine transaminase) and AST (aspartate aminotransferase) activity were also ameliorated after 22B6 mAb treatment (Fig. 4g-h). Furthermore, 22B6 mAb treatment down regulated phosphorylation of Smad2 and JNK in mouse livers compared with the IgG1 group (Fig. 4f). Thus, we deduced that Fstl1 neutralizing antibody could attenuate CCl_4_-induced liver fibrosis in mice through blocking phosphorylation of Smad2/JNK.

We further investigated whether blocking Fstl1 signaling with a neutralizing antibody (22B6 mAb) would inhibit TGF-β1 induced activation of HSCs. We found that HSCs activation marker α-SMA was down regulated after 22B6 mAb treatment (Figure S5a-b). The activation of TGF-β1 signaling measured by phosphorylated Smad2 and JNK were reversed by the 22B6 treatment (Figure S5a, c-d). We also found that 22B6 mAb could significantly prevent TGF-β1 induced ECM production and cell migration compared to isotype matched IgG (IgG1) controls (Fig. S5e-h). Besides, 22B6 mAB significantly decreased chemokine CCL2 and CXCL8 concentrations in cell culture medium compared with IgG (Fig. S5i-j). These data indicate that blocking Fstl1 signaling inhibits TGF-β induced HSCs activation, ECM production and cell migration through inhibiting p-Smad2/JNK.

### TGFβ1-*miR29a*-Fstl1 regulatory circuit in HSCs

**Fig. 5:**
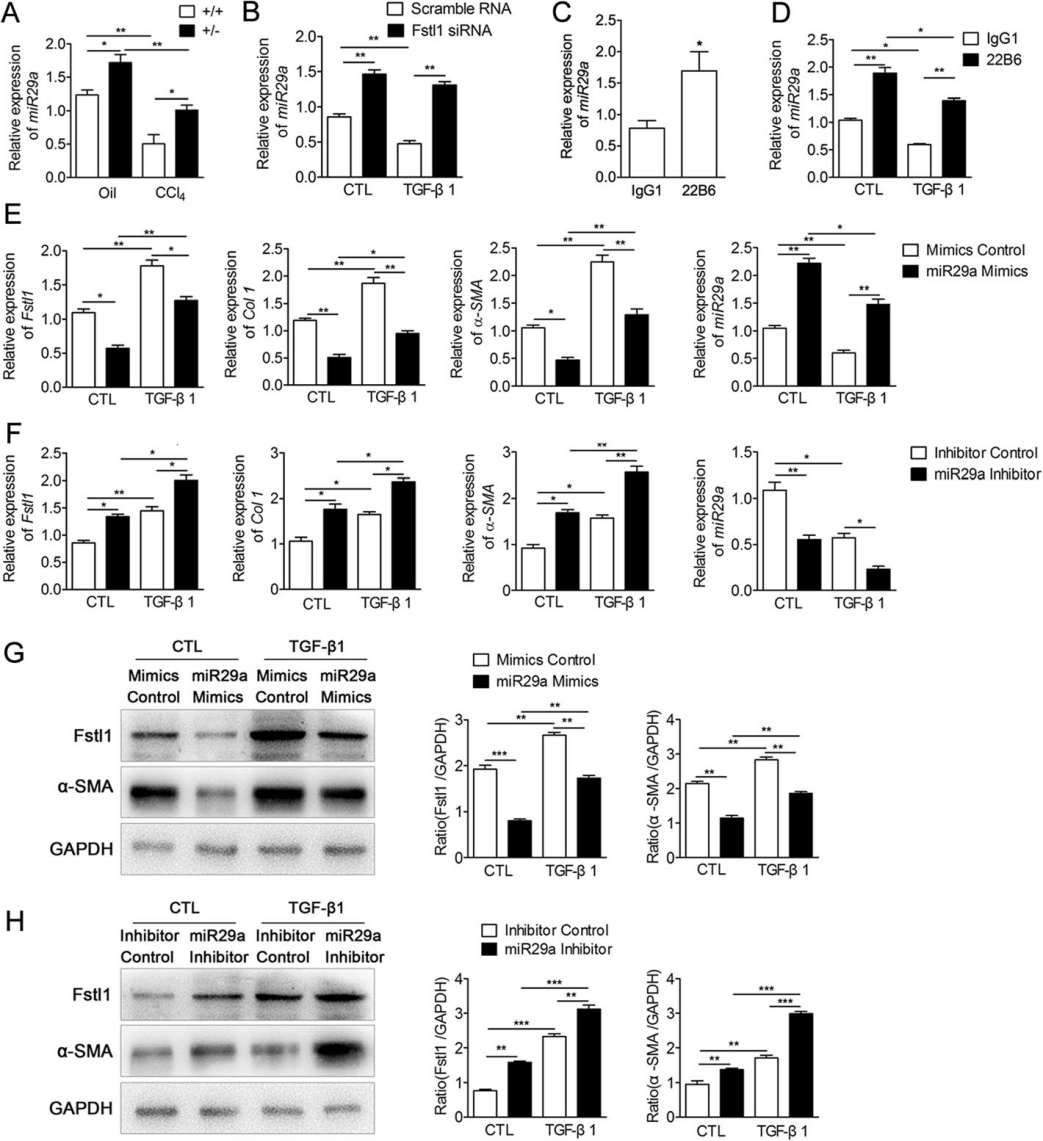
TGF-β1-*miR29a*-Fstl1 Regulatory Circuit Mediates Fibrosis. **a** The livers of *Fstl1*^*+/−*^ mice were harvested for the analyses. The expression of *miR29a* was assessed by qRT-PCR (n = 3 per group). **b** Fstl1 siRNA (40 nM) transfection was performed on LX-2 cells using Lipofectamine RNAimax (n = 3 per group). The expression of *miR29a* was assessed by qRT-PCR (n = 3 per group). **c** BABL/c mice were subjected to CCl_4_ along with Fstl1-neutralizing antibody 22B6 or isotype control IgG1 (25 μg/mouse) twice a week, and livers were harvested 4 weeks later for the analyses. The expression of *miR29a* was assessed by qRT-PCR (n = 6 per group). **d** LX-2 cells were treated with 22B6 or isotype control IgG1(2 μg/ml). The expression of *miR29a* was assessed by qRT-PCR (n = 3 per group). **e** *MiR29a* mimics (100 nM) were transiently transfected into LX-2 cells for 48 h. The expression of *Fstl1*, *Col1*, *α-SMA* mRNA and *miR29a* were assessed by qRT-PCR (n = 3 per group). **f** *MiR29a* inhibitors (200 nM) were transiently transfected into LX-2 cells for 48 h. The expression of *Fstl1*, *Col1*, *α-SMA* mRNA and *miR29a* were assessed by qRT-PCR (n = 3 per group). **g-h** Protein expression levels of α-SMA and Fstl1 in cell extracts were assessed by Western blot. Band intensity was quantified using Image J software and expressed as relative intensity compared with control. The ratio of Fstl1 and α-SMA were subjected to GAPDH (*n* = 2 per group). Throughout, data represent means ± SEM. *P < 0.05, **P < 0.01, ***P < 0.001

Consistent with previous studies [16, 17], we found *miR29a* was down-regulated in response to TGF-β stimulation (Figure S2d). Then we explored the relationship among Fstl1, *miR29a*, and TGF-β1 in liver fibrosis. *Fstl1*^*+/−*^ mice had significant more expression of *miR29a* (Fig. 5a). Fstl1 siRNA significantly increased gene expression of *miR29a* (Fig. 5b), which was independent of TGF-β1 stimulation. Whereas blocking Fstl1 signaling through 22B6 mAb up regulated *miR29a* expression in CCl_4_ treated mice and LX-2 cell line (Fig. 5c-d). Thus, Fstl1 signaling inhibited *miR29a* expression in HSCs in vitro and in liver fibrosis induced by CCl_4_ in vivo.

To determine if *miR29a* direct regulates FSTL1, we used Targetscan to predict consequential pairing of FSTL1 3’UTR target region and subcloned the FSTL1-WT and FSTL1-Mut to the luciferase Open reading frame. The result showed that *miR29a* mimics could suppress luciferase expression when co-transfected with FSTL1-WT plasmid (Figure S6). We also found that *miR29a* mimics transfection significantly down regulated Fstl1 expression, confirming Fstl1 is a *miR29a* target. *MiR-29a* mimics also significant down-regulated gene expression of *Col1*, a known direct target of miR29 [16] and α-SMA (Fig. 5e,g). In consistent with this, *miR29a* inhibitor up-regulated expression of Fstl1, α-SMA and *Col1* (Fig. 5f,h).

To test whether TGF-β1 induces Fstl1 through down regulation of *miR29a*, we used *miR29a* mimics or inhibitor in TGF-β1 stimulated human LX-2 cells. TGF-β1 induced Fstl1, α-SMA and Col1 expression, whereas decreased *miR29a* expression (Fig. 5d-h). *MiR-29a* mimics partially blocked, while *miR29a* inhibitor further enhanced TGF-β1 induced Fstl1 expression (Fig. 5d-h). All together, these data suggest that TGF-β1 might induce Fstl1 partially through down regulation of *miR29a,* while Fstl1 and *miR29*a reciprocally regulate each other in HSCs.

## Discussion

This work highlights the importance of Fstl1 in liver fibrosis. Fstl1 was up regulated in human and mouse fibrotic livers. In the genetic models used, we demonstrated that Fstl1-haplodeficiency mice were less susceptible to chemically induced liver fibrosis. Knockdown Fstl1 significantly inhibited TGF-β1 stimulated cell migration and ECM accumulation. Fstl1-neutraliztion antibody had anti-fibrotic effect in vivo and inhibited HSCs activation and migration in vitro through inhibiting p-Smad2/JNK. Furthermore, we demonstrated that there was a TGF-β1-*miR29a*-Fstl1 regulatory circuit mediating liver fibrosis. These results are in agreement with previous studies showing Fstl1 was induced in liver fibrosis by CCl_4_ treatment [40]. Our observation also confirmed the previous studies that knockdown Fstl1 attenuate liver fibrosis [25, 26].

TGF-β signaling plays a critical role in the regulation of cell growth, migration and differentiation and is a central driver in liver fibrosis [1]. Classical TGF-β signaling is initiated with ligand-induced oligomerization of serine/threonine receptor kinases and phosphorylation of downstream cytoplasmic signaling molecules Smad2 and Smad3 [41, 42]. TGF-β signaling can also affect Smad-independent pathways, such as the MAPK and Akt signaling pathways [4, 41, 43, 44]. Fstl1 was shown as a pro-migratory factor enhancing ERK phosphorylation and regulated by miR-198 in wound healing [45]. Our results implicate that blocking Fstl1 by neutralizing antibody in mice with CCl_4_ treatment also modulates the Smad2 and JNK signaling pathways.

Inflammation is typically present in all chronic liver diseases and associated with the development of fibrosis [46]. FSTL1 has been identified as a novel proinflammatory protein and systemic administration of adenoviral vectors expressing Fstl1 (Ad-Fstl1) to mice induced expression of proinflammatory cytokines in liver and exacerbated collagen-induced arthritis [47]. Conversely, adenovirus-mediated administration of Fstl1 to WT mice with subtotal nephrectomy ameliorated tubulointerstitial fibrosis and reduced expression of proinflammatory mediators in the remnant kidney [22]. 4-methylumbelliferone, an inhibitor of hyaluronan deposition, suppressed the HSC trans-differentiation and altered macrophage localization with the down-regulation of Fstl1 in CCl_4_ treated mice [40]. Further studies are needed to determine whether Fstl1 modulates inflammatory responses in liver fibrosis.

Overexpressing miR-29a/b markedly reduced the degree of liver fibrosis induced by CCl_4_ in mice and decreased collagen expression in LX-2 cells [10, 48]. Ectopic expression of *miR-29b* in activated HSCs also blunted the increased expression of α-SMA, caused cell cycle arrest, and induced apoptosis through targeting PI3K/AKT pathway [11]. Furthermore, Knockout *miR29* enhanced mortality and the expression of fibrotic markers in mouse livers with CCl_4_ treatment [49]. Consistent with this, our studies found *miR29a* mimics significantly decreased COL1, α-SMA and FSTL1 expression, while *miR29a* inhibitors showed the reverse effects. On the other hand, knockdown Fstl1 in LX-2 significantly increased expression of *miR29a*. We also proved that *miR29a* directly targeted FSTL1 3’UTR. Due to sequence similarity, *miR29b* and *miR29c* might also target Fstl1 3’UTR. Blocking Fstl1 signaling through 22B6 mAb up regulated *miR29a* expression in CCl_4_ treated mice. These data suggest that Fstl1 modulates Smad/JNK phosphorylation and *miR29a* in HSC in vitro and in CCl_4_ induced liver fibrosis in mice. There is also a cross talk between Fstl1 and *miR29a* signaling.

We acknowledge several limitations. FSTL1 is a secreted protein which may interact with various extracellular molecules or transmembrane receptors. FSTL1 stimulated the survival and migration of endothelial cells through the cell surface receptor of DIP2A [50]. FSTL1 directly interacted with the secreted phosphoprotein 1 (SPP1)/osteopontin and led to inactivation of integrin/CD44-associated signaling [51]. So Fstl1 may also act on endothelial cells or other cell types in liver fibrosis. Moreover, the mechanisms by which Fstl1 modulates *miR29a* expression remain unresolved.

## Conclusion

Here, we provide evidence that targeting Fstl1 inhibits the activation of HSCs and ameliorates CCl_4_-induced liver fibrosis in mice by modulating TGF-β1-*miR29a*-Fstl1 regulatory circuit and downstream Smad2/JNK signaling in activated HSCs. Fstl1 may serve as a novel therapeutic approach in the treatment for patients with severe liver fibrosis.

## Supplementary information

**Additional file 1 : Table S1.** Characteristics and serum Follistatin-like protein 1 (FSTL1) levels of subjects investigated. **Figure S1.** *FSTL1* Expression in Human Activated HSCs and Senescent HSCs. **Figure S2.** TGF-β1 Induced Fstl1 gene expression in a time-and dose-dependent manner and downregulated miR29a in human LX-2 cell Line. **Figure S3.** TGF-Β1 Induced Fstl1 Gene Expression in a time-and dose-dependent manner in rat CFSC-8B cell line. **Figure S4.** Knockdown of Fstl1 attenuated the activation of LX-2 cells. **Figure S5.** Fstl1-neutralizing antibody reduced LX-2 cell migration, chemokine secretion and inhibiting TGF-β1/Smad2/JNK Signaling. **Figure S6.** MiR29a targets Fstl1 3’UTR.

## Data Availability

All data generated or analyzed during this study are included either in this article or in the supplementary Materials and Methods, Tables, Figures and Figure Legends files.

## Abbreviations

HSCs: Hepatic stellate cells
Fstl1: Follistatin like 1
TGF-β1: Transforming growth factor-β1
ECM: Extracellular matrix
miR29a: microRNA-29a
NAFLD: Nonalcoholic fatty liver disease
Fst: Follistatin
SPARC: Secreted protein acidic rich in cysteines
CCl_4_: Carbon tetrachloride
α-SMA: α-smooth muscle actin
Col1: Collagen I
CCL2: C-C Motif Chemokine Ligand 2
CXCL8: C-X-C Motif Chemokine Ligand 8
PSR: Picro-sirius red
qRT-PCR: Real-time quantitative polymerase chain reaction
ALT: Alanine aminotransferase
AST: Aspartate aminotransferase
ANOVA: one-way analysis of variance
CTL: Control
HBV: Viral hepatitis B
LC: Cirrhosis
HCC: Hepatocellular carcinoma
LSECs: Liver sinusoidal endothelial cells

## References

[1] Bataller R, Brenner DA (2005). Liver fibrosis. J Clin Investig.

[2] Tsuchida T, Friedman SL (2017). Mechanisms of hepatic stellate cell activation. Nat Rev Gastroenterol Hepatol.

[3] Czaja AJ (2014). Hepatic inflammation and progressive liver fibrosis in chronic liver disease. World J Gastroenterol.

[4] Gressner AM, Weiskirchen R, Breitkopf K, Dooley S (2002). Roles of TGF-beta in hepatic fibrosis. Front Biosci.

[5] De Minicis S, Seki E, Uchinami H, Kluwe J, Zhang Y, Brenner DA (2007). Gene expression profiles during hepatic stellate cell activation in culture and in vivo. Gastroenterology..

[6] Seki E, De Minicis S, Osterreicher CH, Kluwe J, Osawa Y, Brenner DA (2007). TLR4 enhances TGF-beta signaling and hepatic fibrosis. Nat Med.

[7] Kanzler S, Lohse AW, Keil A, Henninger J, Dienes HP, Schirmacher P (1999). TGF-beta1 in liver fibrosis: an inducible transgenic mouse model to study liver fibrogenesis. Am J Phys.

[8] Liu X, Hu H, Yin JQ (2006). Therapeutic strategies against TGF-beta signaling pathway in hepatic fibrosis. Liver Int.

[9] Kriegel AJ, Liu Y, Fang Y, Ding X, Liang M (2012). The miR-29 family: genomics, cell biology, and relevance to renal and cardiovascular injury. Physiol Genomics.

[10] Roderburg C, Urban GW, Bettermann K, Vucur M, Zimmermann H, Schmidt S (2011). Micro-RNA profiling reveals a role for miR-29 in human and murine liver fibrosis. Hepatology..

[11] Wang J, Chu ES, Chen HY, Man K, Go MY, Huang XR (2015). microRNA-29b prevents liver fibrosis by attenuating hepatic stellate cell activation and inducing apoptosis through targeting PI3K/AKT pathway. Oncotarget..

[12] Cushing L, Kuang PP, Qian J, Shao FZ, Wu JJ, Little F (2011). miR-29 is a major regulator of genes associated with pulmonary fibrosis. Am J Resp Cell Mol.

[13] Xie T, Liang J, Geng Y, Liu N, Kurkciyan A, Kulur V, et al. MicroRNA-29c prevents pulmonary fibrosis by regulating epithelial cell renewal and apoptosis. Am J Respir Cell Mol Biol. 2017. 10.1165/rcmb.2017-0133OC PMC5765420 28799781

[14] van Rooij E, Sutherland LB, Thatcher JE, DiMaio JM, Naseem RH, Marshall WS (2008). Dysregulation of microRNAs after myocardial infarction reveals a role of miR-29 in cardiac fibrosis. P Natl Acad Sci USA.

[15] Zhang Y, Wu L, Wang Y, Zhang M, Li L, Zhu D (2012). Protective role of estrogen-induced miRNA-29 expression in carbon tetrachloride-induced mouse liver injury. J Biol Chem.

[16] Hambrock HO, Kaufmann B, Muller S, Hanisch FG, Nose K, Paulsson M (2004). Structural characterization of TSC-36/Flik: analysis of two charge isoforms. J Biol Chem.

[17] Shibanuma M, Mashimo J, Mita A, Kuroki T, Nose K (1993). Cloning from a mouse osteoblastic cell line of a set of transforming-growth-factor-beta 1-regulated genes, one of which seems to encode a follistatin-related polypeptide. Eur J Biochem.

[18] Patella S, Phillips DJ, Tchongue J, de Kretser DM, Sievert W (2006). Follistatin attenuates early liver fibrosis: effects on hepatic stellate cell activation and hepatocyte apoptosis. Am J Physiol Gastrointest Liver Physiol.

[19] Camino AM, Atorrasagasti C, Maccio D, Prada F, Salvatierra E, Rizzo M (2008). Adenovirus-mediated inhibition of SPARC attenuates liver fibrosis in rats. J Gene Med.

[20] Geng Y, Dong Y, Yu M, Zhang L, Yan X, Sun J (2011). Follistatin-like 1 (Fstl1) is a bone morphogenetic protein (BMP) 4 signaling antagonist in controlling mouse lung development. Proc Natl Acad Sci U S A.

[21] Dong YY, Geng Y, Li L, Li XH, Yan XH, Fang YS (2015). Blocking follistatin-like 1 attenuates bleomycin-induced pulmonary fibrosis in mice. J Exp Med.

[22] Hayakawa S, Ohashi K, Shibata R, Kataoka Y, Miyabe M, Enomoto T (2015). Cardiac myocyte-derived follistatin-like 1 prevents renal injury in a subtotal nephrectomy model. J Am Soc Nephrol.

[23] Wei K, Serpooshan V, Hurtado C, Diez-Cunado M, Zhao M, Maruyama S (2015). Epicardial FSTL1 reconstitution regenerates the adult mammalian heart. Nature..

[24] Wu Y, Zhou S, Smas CM (2010). Downregulated expression of the secreted glycoprotein follistatin-like 1 (Fstl1) is a robust hallmark of preadipocyte to adipocyte conversion. Mech Dev.

[25] Vollmann EH, Cao L, Amatucci A, Reynolds T, Hamann S, Dalkilic-Liddle I (2017). Identification of novel fibrosis modifiers by in vivo siRNA silencing. Mol Ther Nucleic Acids.

[26] Shang H, Liu X, Guo H (2017). Knockdown of Fstl1 attenuates hepatic stellate cell activation through the TGFbeta1/Smad3 signaling pathway. Mol Med Rep.

[27] Galimov A, Hartung A, Trepp R, Mader A, Fluck M, Linke A (2015). Growth hormone replacement therapy regulates microRNA-29a and targets involved in insulin resistance. J Mol Med (Berl).

[28] Ma R, Wang M, Gao S, Zhu L, Yu L, Hu D, et al. miR-29a Promotes the Neurite Outgrowth of Rat Neural Stem Cells by Targeting Extracellular Matrix to Repair Brain Injury. Stem Cells Dev. 2020;29(9):599–614. 10.1089/scd.2019.0174 31885334

[29] Iredale JP (2007). Models of liver fibrosis: exploring the dynamic nature of inflammation and repair in a solid organ. J Clin Invest.

[30] Jiang D, Liang J, Hodge J, Lu B, Zhu Z, Yu S (2004). Regulation of pulmonary fibrosis by chemokine receptor CXCR3. J Clin Invest.

[31] Mederacke I, Dapito DH, Affo S, Uchinami H, Schwabe RF (2015). High-yield and high-purity isolation of hepatic stellate cells from normal and fibrotic mouse livers. Nat Protoc.

[32] Yang W, Wu Y, Wang C, Liu Z, Xu M, Zheng X (2017). FSTL1 contributes to tumor progression via attenuating apoptosis in a AKT/GSK-3beta - dependent manner in hepatocellular carcinoma. Cancer Biomark.

[33] Moylan CA, Pang H, Dellinger A, Suzuki A, Garrett ME, Guy CD (2014). Hepatic gene expression profiles differentiate presymptomatic patients with mild versus severe nonalcoholic fatty liver disease. Hepatology..

[34] Azimifar SB, Nagaraj N, Cox J, Mann M (2014). Cell-type-resolved quantitative proteomics of murine liver. Cell Metab.

[35] Xu L, Hui AY, Albanis E, Arthur MJ, O'Byrne SM, Blaner WS (2005). Human hepatic stellate cell lines, LX-1 and LX-2: new tools for analysis of hepatic fibrosis. Gut..

[36] Krizhanovsky V, Yon M, Dickins RA, Hearn S, Simon J, Miething C (2008). Senescence of activated stellate cells limits liver fibrosis. Cell..

[37] Seki E, De Minicis S, Gwak GY, Kluwe J, Inokuchi S, Bursill CA (2009). CCR1 and CCR5 promote hepatic fibrosis in mice. J Clin Invest.

[38] Holt AP, Haughton EL, Lalor PF, Filer A, Buckley CD, Adams DH (2009). Liver myofibroblasts regulate infiltration and positioning of lymphocytes in human liver. Gastroenterology..

[39] Zimmermann HW, Seidler S, Gassler N, Nattermann J, Luedde T, Trautwein C (2011). Interleukin-8 is activated in patients with chronic liver diseases and associated with hepatic macrophage accumulation in human liver fibrosis. PLoS One.

[40] Andreichenko IN, Tsitrina AA, Fokin AV, Gabdulkhakova AI, Maltsev DI, Perelman GS, et al. 4-methylumbelliferone Prevents Liver Fibrosis by Affecting Hyaluronan Deposition, FSTL1 Expression and Cell Localization. Int J Mol Sci. 2019;20(24):6301. 10.3390/ijms20246301 PMC6941058 31847129

[41] Lechuga CG, Hernandez-Nazara ZH, Dominguez Rosales JA, Morris ER, Rincon AR, Rivas-Estilla AM (2004). TGF-beta1 modulates matrix metalloproteinase-13 expression in hepatic stellate cells by complex mechanisms involving p38MAPK, PI3-kinase, AKT, and p70S6k. Am J Physiol Gastrointest Liver Physiol.

[42] Meng XM, Tang PM, Li J, Lan HY (2015). TGF-beta/Smad signaling in renal fibrosis. Front Physiol.

[43] Koren J, Jinwal UK, Jin Y, O'Leary J, Jones JR, Johnson AG (2010). Facilitating Akt clearance via manipulation of Hsp70 activity and levels. J Biol Chem.

[44] Wang Y (2008). Phosphatidylinositol 3-kinase/Akt pathway regulates hepatic stellate cell apoptosis. World J Gastroenterol.

[45] Sundaram GM, Ismail HM, Bashir M, Muhuri M, Vaz C, Nama S, et al. EGF hijacks miR-198/FSTL1 wound-healing switch and steers a two-pronged pathway toward metastasis. J Exp Med. 2017. 10.1084/jem.20170354 PMC5626400 28827448

[46] Seki E, Schwabe RF (2015). Hepatic inflammation and fibrosis: functional links and key pathways. Hepatology..

[47] Miyamae T, Marinov AD, Sowders D, Wilson DC, Devlin J, Boudreau R (2006). Follistatin-like protein-1 is a novel proinflammatory molecule. J Immunol.

[48] Bian EB, Li J, Zhao B (2014). miR-29, a potential therapeutic target for liver fibrosis. Gene..

[49] Kogure T, Costinean S, Yan I, Braconi C, Croce C, Patel T (2012). Hepatic miR-29ab1 expression modulates chronic hepatic injury. J Cell Mol Med.

[50] Ouchi N, Asaumi Y, Ohashi K, Higuchi A, Sono-Romanelli S, Oshima Y (2010). DIP2A functions as a FSTL1 receptor. J Biol Chem.

[51] Chiou J, Chang YC, Tsai HF, Lin YF, Huang MS, Yang CJ (2019). Follistatin-like protein 1 inhibits lung Cancer metastasis by preventing Proteolytic activation of Osteopontin. Cancer Res.

