# Supplementary material for: Targeting Follistatin like 1 ameliorates liver fibrosis induced by carbon tetrachloride through TGF-β1-miR29a in mice
Source: Cell Commun Signal. 2020 Sep 15;18:151. doi: 10.1186/s12964-020-00610-0 (PMC7493388; doi:10.1186/s12964-020-00610-0)
Supplement: Supplementary file 2 — Additional file 1 : Table S1. Characteristics and serum Follistatin-like protein 1 (FSTL1) levels of subjects investigated. Figure S1. FSTL1 Expression in Human Activated HSCs and Senescent HSCs. Figure S2. TGF-β1 Induced Fstl1 gene expression in a time-and dose-dependent manner and downregulated miR29a in human LX-2 cell Line. Figure S3. TGF-Β1 Induced Fstl1 Gene Expression in a time-and dose-dependent manner in rat CFSC-8B cell line. Figure S4. Knockdown of Fstl1 attenuated the activation of LX-2 cells. Figure S5. Fstl1-neutralizing antibody reduced LX-2 cell migration, chemokine secretion and inhibiting TGF-β1/Smad2/JNK Signaling. Figure S6. MiR29a targets Fstl1 3’UTR. [file 12964_2020_610_MOESM2_ESM.docx]

**Supporting Table 1: Characteristics and serum Follistatin-like protein 1 (FSTL1) levels of subjects investigated**

| **Characteristics** | **Controls** | **Hepatitis B Viral** | **Cirrhosis** | **Hepatocellular carcinoma** |
| --- | --- | --- | --- | --- |
| **Total number of subjects** | 27 | 19 | 4 | 15 |
| **Gender (male/female) (*n*)** | 13/14 | 11/8 | 3/1 | 8/7 |
| **Age (years)** | 73 (61-87) | 54 (31-71) | 43 (32-57) | 56 (31-71) |
| **25^th^ percentile** | 67 | 44 | 33 | 50 |
| **75^th^ percentile** | 78 | 62 | 51 | 66 |
| **Serum FSTL1 level (ng/mL)** |  |  |  |  |
| **Average** | 6.67 | 15.02 | 18.51 | 14.09 |
| **Geometric mean** | 6.00 | 13.79 | 19.09 | 13.02 |
| **Median** | 5.89 | 12.41 | 16.18 | 12.15 |
| **25^th^ percentile** | 4.43 | 10.22 | 12.16 | 10.01 |
| **75^th^ percentile** | 7.70 | 20.31 | 22.54 | 16.95 |
| ***p* versus Controls** | - | <0.001 | <0.001 | <0.001 |
| **Serum ALT level (U/L)** |  |  |  |  |
| **Average** | 19.60 | 44.71 | 26.95 | 49.44 |
| **Geometric mean** | 17.79 | 33.96 | 24.46 | 37.07 |
| **Median** | 17.10 | 33.50 | 26.35 | 33.50 |
| **25^th^ percentile** | 13.60 | 20.10 | 16.05 | 23.20 |
| **75^th^ percentile** | 22.70 | 58.25 | 37.25 | 67.25 |
| ***p* versus Controls** | - | <0.05 | - | <0.05 |
| **Serum AST level (U/L)** |  |  |  |  |
| **Average** | 21.05 | 62.28 | 40.60 | 68.07 |
| **Geometric mean** | 20.60 | 42.27 | 31.67 | 45.66 |
| **Median** | 19.60 | 31.90 | 23.85 | 34.60 |
| **25^th^ percentile** | 18.00 | 22.90 | 21.63 | 25.75 |
| **75^th^ percentile** | 22.50 | 81.75 | 42.83 | 81.75 |
| ***p* versus Controls** | - | <0.05 | - | <0.05 |

**Supplementary Figure 1**


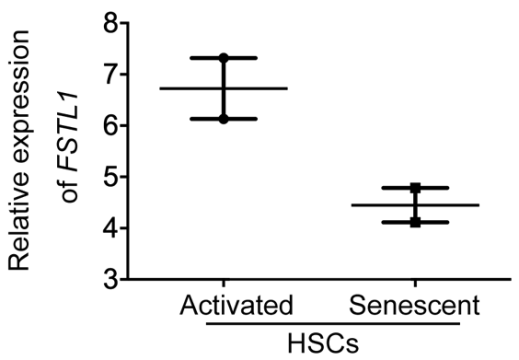


**Figure S1. *FSTL1* Expression in Human Activated HSCs and Senescent HSCs.**

Cell senescence was induced by DNA-damaging agent etoposide. The data were acquired from a published gene-profiling dataset (GEO accession: GDS3492). n=2 per group.

**Supplementary Figure 2**


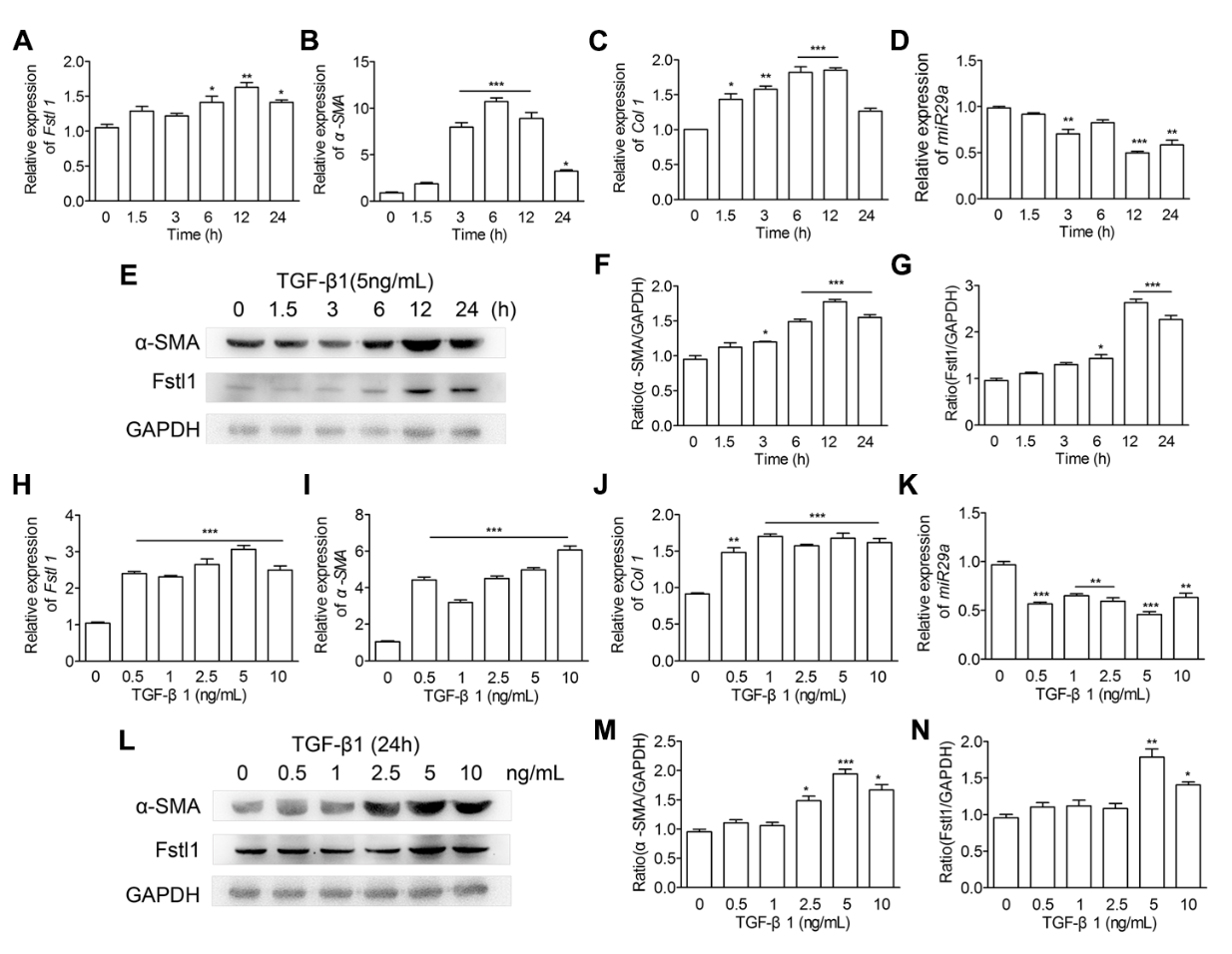


**Figure S2. TGF-β1 Induced Fstl1 gene expression in a time-and dose-dependent manner and downregulated miR29a in human LX-2 cell Line.** (A-G) LX-2 cells were treated with 5 ng/ml TGF-β1 for 0, 1.5, 3, 6, 12, 24h. (H-N) LX-2 cells were treated with TGF-β1(0, 0.5, 1, 2.5, 5, 10 ng/ml) for 24h. The expression of *Fstl1* (A, H)*, α-SMA* (B, I)*, Col1* (C, J) mRNA and *miR29a* (D, K) were assessed by qRT-PCR. (E-G,L-N) Protein expression levels of α-SMA and Fstl1 in cell extracts were assessed by Western blot. GAPDH was used as a loading control. Throughout, data represent mean ± SEM. **P* < 0.05, ***P* < 0.01, ****P* < 0.001 versus control group.

**Supplementary Figure 3**


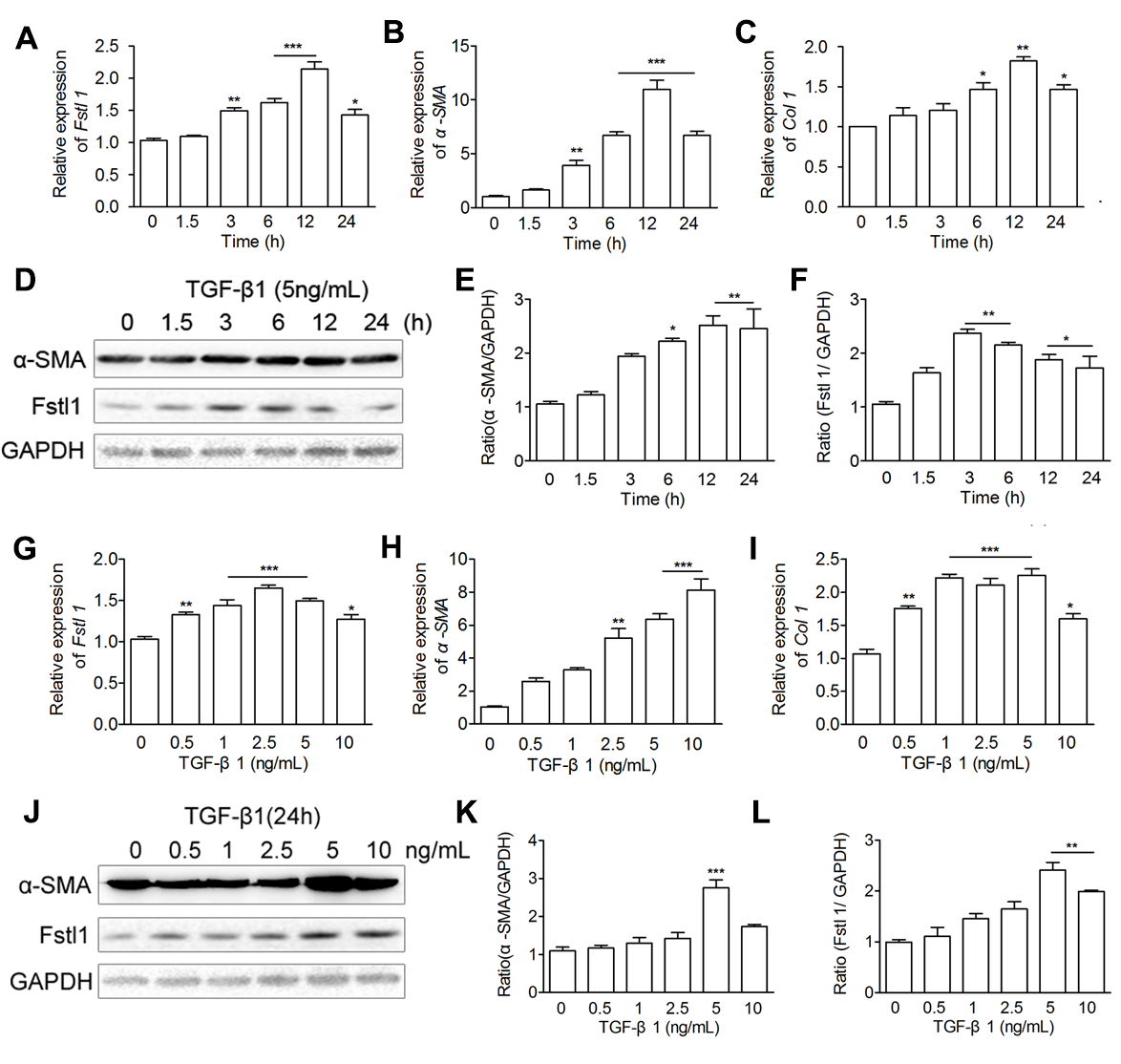


**Figure S3. TGF-Β1 Induced Fstl1 Gene Expression in a time-and dose-dependent manner in rat CFSC-8B cell line.** (A-F) CFSC-8B cells were treated with 5 ng/ml TGF-β1 for 0, 1.5, 3, 6, 12, 24h. (G-L) CFSC-8B cells were treated with TGF-β1 (0, 0.5, 1, 2.5, 5, 10 ng/ml) for 24h. The expression of *Fstl1* (A, G)*, α-SMA* (B, H)*, Col1* (C, I) mRNA were assessed by qRT-PCR. (D-F, J-L) Protein expression levels of α-SMA and Fstl1 in cell extracts were assessed by Western blot. GAPDH was used as a loading control. Throughout, data represent mean ± SEM. **P* < 0.05, ***P* < 0.01, ****P* < 0.001 versus control group.

**Supplementary Figure 4**

**
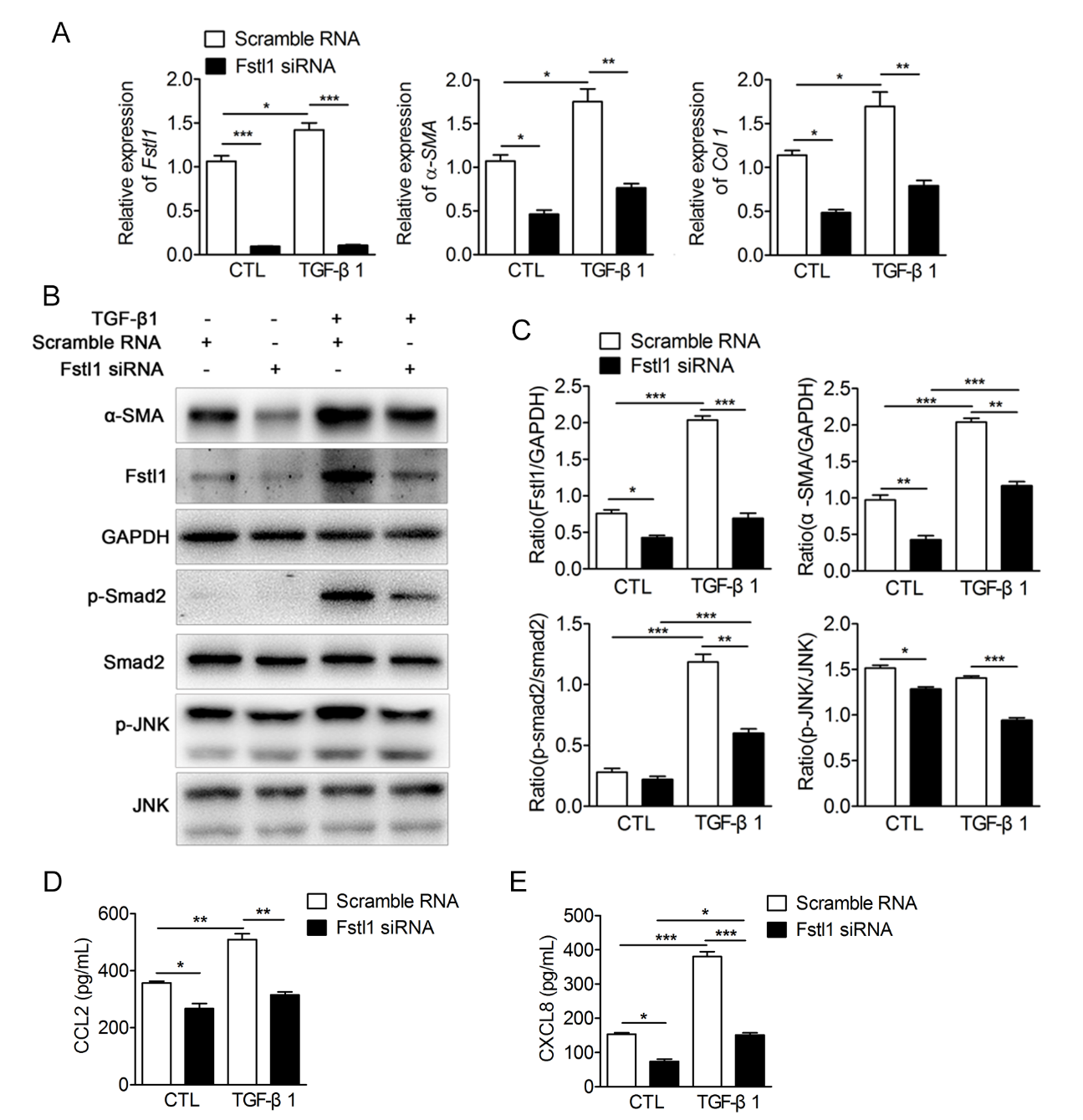
**

**Figure S4. Knockdown of Fstl1 attenuated the activation of LX-2 cells.** (A) Fstl1 siRNA (40nM) transfection was performed on LX-2 cells using Lipofectamine RNAimax. The gene expressions of *Fstl1*, *α-SMA*, *Col1* mRNA were assessed by qRT-PCR (n = 3 per group). (B) α-SMA, Fstl1, p-Smad2, Smad2, p-JNK, JNK were analyzed by Western blot (n = 2 per group). (C) Band intensity was quantified using Image J software and expressed as relative intensity compared with control. The ratio of Fstl1, α-SMA were subjected to GAPDH. The ratio of p-Smad2 was subjected to Smad2. The ratio of p-JNK was subjected to JNK. Scramble RNA was used as control. CCL2 (D) and CXCL8 (E) concentration in the cell culture medium (n = 3 per group). Throughout, data represent mean ± SEM. **P*< 0.05, ***P*< 0.01, ****P*< 0.001.

**Supplementary Figure 5**


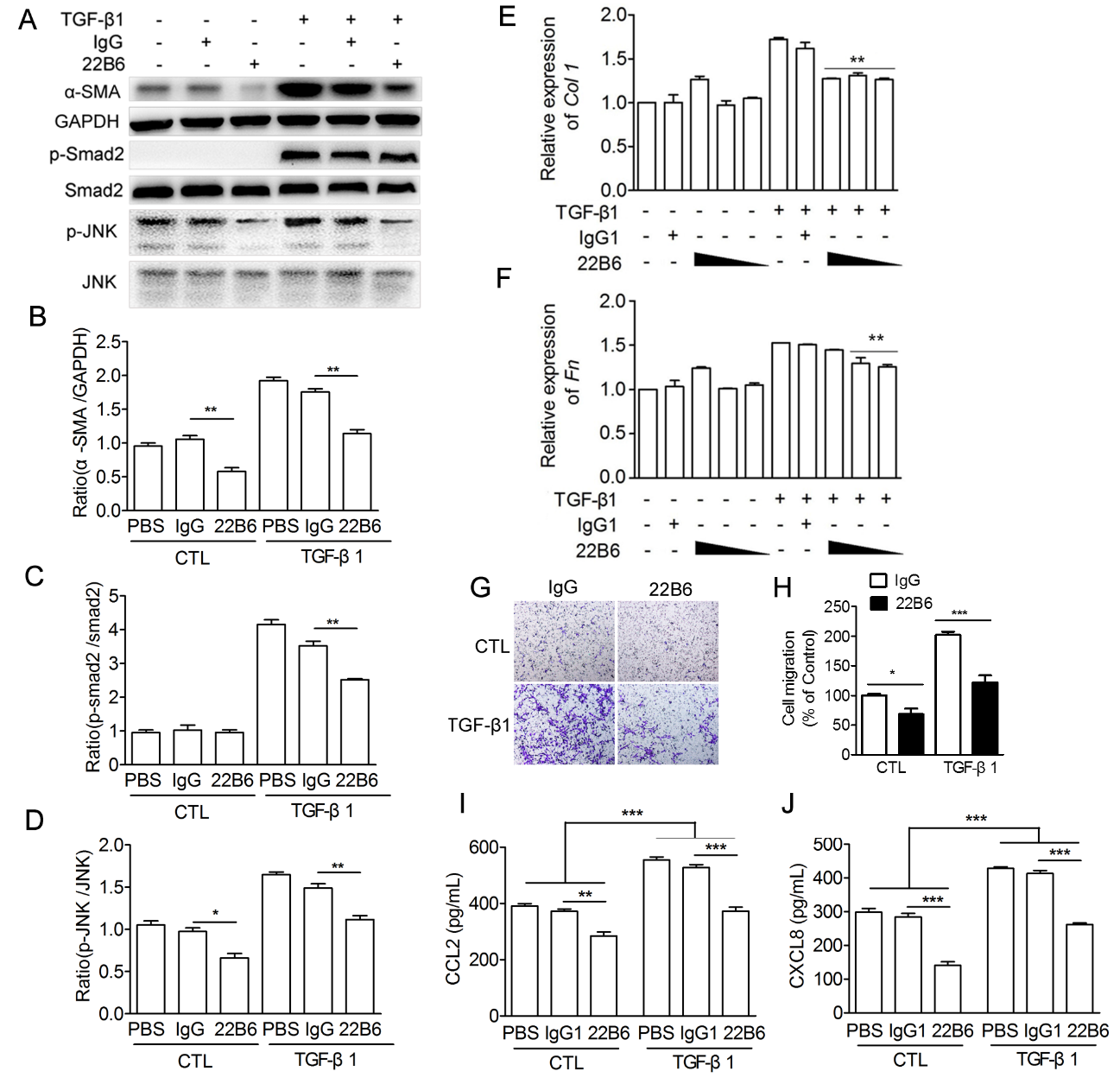


**Figure S5.** **Fstl1-neutralizing antibody reduced LX-2 cell migration, chemokine secretion and inhibiting TGF-β1/Smad2/JNK Signaling.** (A) Cells were pretreated with 2 µg/ml antibody (22B6) or control IgG1 for 24 h and then treated with 5 ng/ml TGF-β1. The α-SMA, GAPDH, p-Smad2, Smad2, p-JNK and JNK protein expressions were analyzed by Western blot. (B-D) Band intensity was quantified using Image J software and expressed as relative intensity compared with control. The ratio of α-SMA were subjected to GAPDH. The ratio of p-Smad2 was subjected to Smad2. The ratio of p-JNK was subjected to JNK. (E,F) The RNA expression level of Col1 and Fn were accessed by qRT-PCR after 1, 2, 4 µg/ml antibody (22B6) treatment. (G,H) The migration of LX-2 was measured using the transwell system. Transmigration was evaluated 24h after seeding the cells, by counting crystal violet-staining cells on the underside membrane by light microscopy. Images were photographed at 100 amplifications. CCL2 (I) and CXCL8 (J) concentration in the cell culture medium (n = 3 per group). Throughout, data represent mean ± SEM. *p < 0.05, **p < 0.01, ***p < 0.001 between two groups.


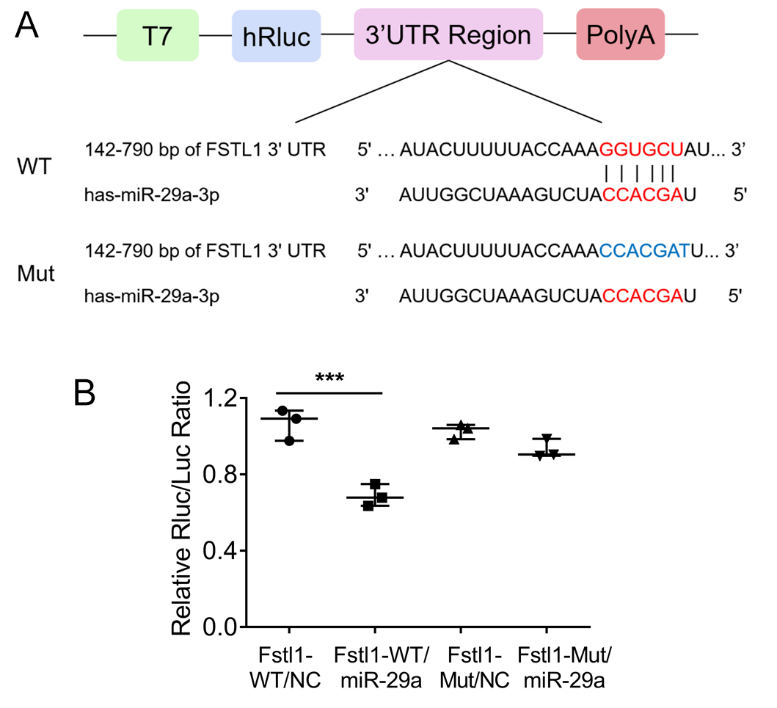
**Supplementary Figure 6**

**Figure S6. MiR29a targets Fstl1 3’UTR.** (A) MiR29a binding site in FSTL1 3’UTR and the design of luciferase report plasmids. (B) Luciferase activity of LX-2 cells transfected with a reporter plasmid containing a Renilla luciferase (hRluc) with the Fstl1-WT and Fstl1-Mutant 3′ UTR. All data were normalized to firefly luciferase gene (hLuc) activity. n = 3, ***P < 0.001.
